# Editorial: Contemporary challenges in diagnosis and treatment of predominantly antibody deficiency

**DOI:** 10.3389/fimmu.2022.959720

**Published:** 2022-08-15

**Authors:** Rohan Ameratunga, Hassan Abolhassani, Paul J. Maglione, Emily S. J. Edwards

**Affiliations:** ^1^ Department of Clinical immunology, Auckland Hospital, Auckland, New Zealand; ^2^ Department of Virology and Immunology, Auckland Hospital, Auckland, New Zealand; ^3^ Department of Molecular Medicine and Pathology, School of Medicine, Faculty of Medical and Health Sciences, University of Auckland, Auckland, New Zealand; ^4^ Department of Biosciences and Nutrition, Karolinska University Hospital, Karolinska Institutet, Huddinge, Sweden; ^5^ Pulmonary Center and Section of Pulmonary, Allergy, Sleep & Critical Care, Department of Medicine, Boston University School of Medicine, Boston, MA, United States; ^6^ Allergy and Clinical Immunology Laboratory, Department of Immunology and Pathology, Monash University, Melbourne, VIC, Australia

**Keywords:** predominantly antibody deficiencies, diagnostic challenges, treatment challenges, antibody defects, cellular defects

Primary immunodeficiencies (PID) otherwise referred to as Inborn Errors of Immunity (IEI) are rare inherited diseases of the immune system. According to the International Union of Immunological Societies (IUIS) classification, predominantly antibody deficiency (PAD) is the most common PID, characterised by low serum immunoglobulin levels, poor vaccination responses and a high incidence of infectious and non-infectious complications including cancer. In adults, IgA deficiency (IgAD) and Common Variable Immunodeficiency Disorders (CVID) comprise the majority of these conditions. Diverse clinical, immunological, and genetic phenotypes in PAD result in diagnostic delays and poor access to targeted treatments, accounting for the early mortality and high morbidity of this population. The current series of fourteen articles on Contemporary Challenges in Diagnosis and Treatment of PAD explores aspects of the diagnosis and treatment of of this group of conditions. Here, the editors of this section summarise the main findings of the articles in this series.

## Diagnosis and semantics

The first article in the series explores the terminology and semantics of this group of disorders (Ameratunga et al.) In recent years there has been a move to rename these conditions as Inborn Errors of Immunity (IEI) rather than PIDs. However, the majority of patients with PIDs do not yet have a genetic explanation. These disorders include CVID (by definition), IgAD, transient hypogammaglobulinemia of infancy (THI) and Good’s syndrome comprising the majority of PIDs. Additionally, not all patients with conditions such as Severe Combined Immunodeficiency have a genetic diagnosis. Therefore, this article argues that at present it is premature to label all patients with PIDs as having IEIs. In the future, diagnostic advances may make it possible to genetically define all of these conditions and then the use of PID and IEI will be interchangeable.

Where feasible, all patients with PIDs should be offered genetic studies. There are many overlapping clinical advantages in securing a genetic diagnosis. It may allow the accurate diagnosis of an atypical presentation of PID, which could permit prenatal or pre-implantation diagnosis, direct prognostic monitoring of patients e.g. monitoring for development of non-infectious complications associated with a particular genetic defect, or improve patient access to targeted therapeutics. In PAD, genetic diagnosis rates are currently low (<20-30% in most nonconsanguineous cohorts). Despite the aforementioned genetically undiagnosed PAD patients, the use of Next Generation Sequencing (NGS) has significantly increased the number of genes shown to underlie PAD. The article by Rojas-Rostrepo et al., presents the findings of NGS in 291 patients with PAD. In 24.7% (72/291) of patients a relevant genetic defect was identified by NGS. It should be noted that some of these patients had gene panels while others had whole exome sequencing. The authors confirm the yield from this study may have been higher if whole exome sequencing had been deployed for all patients, showing the validity of this approach in the diagnostic work up of PAD. A wide variety of genetic mutations were identified, highlighting the genetic complexity of these diseases. Importantly, an impact on protein expression and/or function was proven in these patients (n=72), confirming variant pathogenicity. It should be noted that the diagnostic yield in consanguineous populations is much higher for predominantly autosomal recessive conditions.

In PAD, vaccine responses are characteristically low, and notoriously difficult to assess in patients receiving immunoglobulin replacement therapy. The study by Hansen et al., describes a new approach to assess responses to the pneumococcal vaccine. Currently at least five criteria for assessing responses to Pneumovax^®^ exist. This article examines the use of a Z score to quantify responses to the Pneumococcal vaccine. The advantage of the Z score is that it avoids the dichotomous responses advocated by the American Academy of Asthma Allergy and Immunology. Such an approach distinguishes patients with symptomatic hypogammaglobulinemia from those with milder symptoms. Future studies will indicate if such an approach resolves difficulties with interpreting vaccine challenge responses in antibody deficient patients.

There is often a long lag between the onset of symptoms and identification of hypogammaglobulinemia in antibody deficiency disorders. The study by Piza et al., explores the utility of cost-effective serum electrophoresis and calculated globulin fractions for identification of patients who may have hypogammaglobulinemia. The area under the curve in the gamma region of serum protein electrophoresis (EPG) may prove to be a useful screening tool for earlier diagnosis of hypogammaglobulinemia. This may reduce the diagnostic lag to implement quicker treatment for patients.

## Mechanism of disease

Cytotoxic T-lymphocyte-associated protein 4 (CTLA-4) and programmed cell death protein 1 (PD-1) regulate cellular immunity and represent important targets for immunotherapy. The article by Hao and Cook explores interesting parallels between phenocopies and patients with genetic defects in CTLA-4 or PD-1. Anti-CTLA-4 treatment is used to treat patients with severe autoimmunity and malignancy, whilst checkpoint inhibitors including anti-PD-1 are used to treat cancer, most notably metastatic melanoma. Patients suffering from genetic defects of CTLA-4 and PD-1 are at risk of autoimmunity. The recapitulation of the phenotype by drug treatment confirms the role of these molecules in the pathogenesis of these disorders. There are however interesting differences in the phenotype of CTLA-4 haploinsufficiency, and patients treated with ipilimumab (anti CTLA-4). Most notably patients with CTLA-4 haploinsufficiency had a much greater risk of infections. Similarly, a family with congenital deficiency of PD-1 had similar autoimmune manifestations including type 1 diabetes, as is often seen in patients undergoing treatment with PD-1 checkpoint inhibitors. Some of the differences between the congenital versions of these disorders and their phenocopies may relate to incomplete inhibition of the pathway and any effect of the Fc component of the antibody used to block the molecule.

Kabir et al., review what is known about Good’s syndrome, a rare, combined immunodeficiency. This disorder is characterised by hypogammaglobulinemia, B cell lymphopenia and a T cell defect in the presence of a thymoma. Moreover, many features overlap with CVID including bacterial infections, and haematological disease, as well as an absence of a genetic explanation for disease. Thus, the authors suggest a more thorough investigation of Good’s syndrome is required to understand the underlying immunopathology and to support a more definitive diagnosis.

## The impact of COVID-19, including vaccines on antibody deficient patients

The COVID-19 pandemic has caused a global crisis. Apart from well over 6 million deaths, large numbers of individuals are suffering from ongoing physical and psychiatric morbidity. In addition, it has raised significant concerns for patients with PAD due to poor immunity to vaccination and increased susceptibility to severe infection with pathogens including viruses. Thus far vaccination and antiviral therapeutics represent the safest options for patients with normal immune systems as well as those suffering from antibody (and other) immunodeficiencies, with triple doses of vaccination recommended as a primary regimen for those with immunodeficiencies (compared to a double primary dose for healthy individuals). Furthermore, it is expected that intravenous or subcutaneous immunoglobulin (SCIG/IVIG) replacement therapy will provide added protection for those antibody deficient recipients.

Ameratunga et al., review the potential utility of vaccines in patients with antibody deficiency. Two recent studies have shown that patients with CVID are able to generate antibodies against tetanus toxoid and Haemophilus influenza type B. This indicates that COVID-19 vaccines will be at least partially effective in patients with CVID. However, the breadth, longevity, and protective capacity of different facets of immunity: antibodies, memory B cells and memory T cells is required to understand whether vaccine-elicited responses to the SARS-CoV-2 virus can protect from breakthrough infection and severe disease. This article highlights the need for individual assessment of patients to determine the degree of immunity generated in response to vaccination, especially in patients receiving SCIG/IVIG where antibody responses cannot be accurately quantified. Moreover, it might be necessary to boost patients without a measurable SARS-CoV-2-specific T cell response every six months, to ensure longevity of the response. However, this will need to be closely monitored to prevent overactivation and exhaustion of the T cell compartment.

In line with this, the article by Quinti et al. explores antibody and cellular responses to COVID-19 vaccines. It appears that patients with CVID generate atypical memory B cells in response to COVID-19 vaccines, which might be short-lived and only develop partial T cell immunity. The authors suggest robust immunity can be induced with post infection-induced vaccination. In contrast, infection produces typical memory B cells, which may be more protective. Here, the authors suggest CVID COVID-19 survivors should have booster vaccines as hybrid immunity may provide long-lasting protection. Furthermore, since reinfection with SARS-CoV-2 has been reported the authors postulate that CVID patients may benefit from preventive therapies such as administration of monoclonal antibodies against the spike protein of SARS-CoV-2.

The response to COVID-19 vaccines in patients with mild antibody defects has not been studied. The study by Sauerwein et al., evaluated antibody and CD4^+^ T cell responses after two doses of the Pfizer BNT162b2 vaccine in 31 adult PAD patients. 76% PAD patients mounted protective SARS-CoV-2-specific IgG responses whereas 87% generated SARS-CoV-2-specific IgA antibodies. The study also showed activation of CXCR5^+^ CD4^+^ T cells after vaccination. Activation of these follicular helper CD4^+^ T cells was associated with the generation of anti-Spike antibodies. It appears patients with mild antibody defects respond to COVID-19 vaccines analogous to normal controls. Patients suffering from CVID had variable CD4^+^ T cell responses, which correlated with the levels of neutralising antibody titres, suggesting an important role for CD4^+^ T cells in vaccine efficacy in CVID. This study attests to the importance of being able to measure T cell responses to COVID-19 and supports that at least two doses of the Pfizer BNT162b2 vaccine were required for vaccine efficacy in this population.

## Complications of antibody deficiency

Infection is the most common complication in PAD. Viral infections are frequently the source of morbidity and mortality in immunodeficient patients. In contrast to the broad array of antibacterial drugs, there are fewer effective antiviral drugs. Some infections such as intractable norovirus can lead to death from nutritional complications. A case series by Chan et al., highlights the role of Cytomegalovirus (CMV) in producing chronic infection in patients with CVID. This cohort had a high mortality attesting to the importance of CMV infections in prognosis and CMV centric therapeutics in patients at risk of CMV-related disease.

Furthermore, an increased risk of malignancy in patients with antibody defects has been identified for several decades. These can either arise spontaneously or following a viral infection. It has been recognised for many years that patients with antibody defects have an increased susceptibility to gastric cancer as well as lymphoid malignancy. Thus, the mechanisms underlying cancer susceptibility are urgently needed for better diagnosis and treatment of this comorbidity. In genetically undiagnosed patients, the study by Bruns et al., shows that 12.3% (27/219) patients had cancer, with gastric cancer, non-Hodgkin’s lymphoma and non-melanoma skin cancer being the most prevalent. Whilst no significant differences in immunological phenotype were observed, a definite or likely genetic diagnosis was identified in 11% of the patients with cancer. Furthermore, it was posed that a likely genetic susceptibility to cancer was identified in 14.3% of patients. This provides important insight into the prevalence of cancer in PAD and the genetic basis of disease, which may contribute to genetic screening for this comorbidity. In contrast, the review by Abolhassani et al., examined the prevalence of cancer in the context of specific monogenic defects. The complex development of cancer was compared between PAD patients and other PID groups, based on 10 hallmarks of cancer. This provides information, which can improve the genetic diagnosis and identification of hallmarks of PAD, which may direct more targeted patient treatment.

Patients with CVID are predisposed to liver disease, most notably nodular regenerative hyperplasia (DiGiacomo et al.). The measurement of transient liver stiffness in patients with CVID is a non-invasive method for identification and monitoring this complication. This study by DiGiacomo et al. shows that this test can be undertaken periodically to diagnose and monitor progress of portal hypertension in these patients to prompt referral to the hepatology clinic.

## Therapy

There is ongoing debate about the merits of subcutaneous vs intravenous immunoglobulin therapy in antibody deficiencies. This article explores patient preferences for SCIG vs IVIG (Gonzalez et al.). It appears there is considerable heterogeneity of preferences in the route of administration. This suggests an integral partnership between physician and patient will enhance the therapeutic relationship, to improve patient care.

## Summary

The past decade has provided improved insights into the molecular and immunological underpinnings of PAD. The articles in this series have covered many areas of ongoing research attesting to the number of groups around the world investigating PAD. This augurs well for patients suffering from these conditions as new diagnostic and therapeutic modalities will result from these endeavours. This will ultimately increase diagnostic rates, reduce the diagnostic delay, and improve patient access to targeted therapies leading to improved patient quality of life and reducing the morbidity of disease.

## Authors contributions

RA and ESJE reviewed the published papers on the Research Topic and wrote the manuscript. All authors critically evaluated, edited, and approved the submitted article.

## Conflict of interest

The authors declare that the research was conducted in the absence of any commercial or financial relationships that could be construed as a potential conflict of interest.

## Publisher’s note

All claims expressed in this article are solely those of the authors and do not necessarily represent those of their affiliated organizations, or those of the publisher, the editors and the reviewers. Any product that may be evaluated in this article, or claim that may be made by its manufacturer, is not guaranteed or endorsed by the publisher.

